# The longitudinal development of social and executive functions in late adolescence and early adulthood

**DOI:** 10.3389/fnbeh.2015.00252

**Published:** 2015-09-15

**Authors:** Sophie J. Taylor, Lynne A. Barker, Lisa Heavey, Sue McHale

**Affiliations:** Department of Psychology, Sociology and Politics, Sheffield Hallam UniversitySheffield, UK

**Keywords:** adolescence, longitudinal, developmental trajectory, social cognition, executive function

## Abstract

Our earlier work suggests that, executive functions and social cognition show protracted development into late adolescence and early adulthood (Taylor et al., 2013). However, it remains unknown whether these functions develop linearly or non-linearly corresponding to dynamic changes to white matter density at these age ranges. Executive functions are particularly in demand during the transition to independence and autonomy associated with this age range (Ahmed and Miller, 2011). Previous research examining executive function (Romine and Reynolds, 2005) and social cognition (Dumontheil et al., 2010a) in late adolescence has utilized a cross sectional design. The current study employed a longitudinal design with 58 participants aged 17, 18, and 19 years completing social cognition and executive function tasks, Wechsler Abbreviated Scale of Intelligence (Wechsler, 1999), Positive and Negative Affect Schedule (Watson et al., 1988), and Hospital Anxiety and Depression Scale (Zigmond and Snaith, 1983) at Time 1 with follow up testing 12–16 months later. Inhibition, rule detection, strategy generation and planning executive functions and emotion recognition with dynamic stimuli showed longitudinal development between time points. Self-report empathy and emotion recognition functions using visual static and auditory stimuli were stable by age 17 whereas concept formation declined between time points. The protracted development of some functions may reflect continued brain maturation into late adolescence and early adulthood including synaptic pruning (Sowell et al., 2001) and changes to functional connectivity (Stevens et al., 2007) and/or environmental change. Clinical implications, such as assessing the effectiveness of rehabilitation following Head Injury, are discussed.

## Introduction

Adolescence is a critical period of development with dynamic brain maturation characterized by psychological, behavioral and social change (Steinberg and Morris, 2001) indicative of the transition to autonomy and independence. Executive functions and socio-emotional development are key to adaptive functioning in this stage of development (Ahmed and Miller, 2011). Executive functions initiate, co-ordinate, maintain, and inhibit other cognitive functions (Miyake et al., 2000) and are recruited in novel or demanding situations to perform goal-directed behavior when routine behavior is inadequate. Social cognition incorporates a range of functions including emotion recognition, empathy, perspective taking, and Theory of Mind (ToM), the ability to impute a range of mental states including beliefs, desires, and intentions to self and others (Frith, 2007; Carrington and Bailey, 2009). During adolescence some cognitive functions show protracted development including updating and switching (Magar et al., 2010), verbal fluency and planning (Romine and Reynolds, 2005), emotion recognition (Thomas et al., 2007), perspective taking (Choudhury et al., 2006; Dumontheil et al., 2010a), and empathy (Mestre et al., 2009). However, these data predominantly focus on younger age ranges with less known about late adolescent and early adulthood development. Furthermore, these studies indicate linear development of cognitive functions whereas there is also contrasting evidence of non-linear development (Taylor et al., 2013).

Findings from imaging studies indicate that brain maturation is dynamic across development including both progressive (myelination) and regressive (synaptic pruning) processes (Sowell et al., 2001) with protracted development of frontal networks into late adolescence and early adulthood (Schmithorst and Yuan, 2010). The continued development of frontal networks is particularly pertinent because they are thought to play an important role in executive functions (Barker et al., 2010) and some aspects of social cognition (Carrington and Bailey, 2009) that are crucial to adaptive goal-oriented behavior. Diffusion Tensor Imaging (DTI) data show protracted maturation of frontal networks (Schmithorst and Yuan, 2010) are associated with the executive function of strategy generation (Delis et al., 2001) between 16.2 and 20.6 years of age at Time 1 with follow-up testing 16 months later (Bava et al., 2010). These findings indicate that white matter maturation in late adolescence and early adulthood leads to an improvement of executive functions and provide the neural basis of developmental change in certain aspects of cognition.

### Executive functions

Late adolescence is characterized by linear and non-linear brain maturation that may correspond behaviorally to functions showing linear or non-linear development, for example troughs and peaks in development (Fischer and Kennedy, 1997). Behavioral studies provide evidence of linear and non-linear executive function development in late adolescence. In a meta-analysis of cross-sectional executive function studies, Romine and Reynolds (2005) reported that executive functions show divergent developmental trajectories with planning and verbal fluency continuing to develop linearly between late adolescence and early adulthood. Magar et al. (2010) provided further support for linear executive function development with updating, assessed with the n-back task (Cohen et al., 1997) and switching, assessed with the number-letter switching task (Rogers and Monsell, 1995), improving between ages 11 and 17.

There is also some evidence of non-linear development with poorer performance on executive function tasks in late adolescence compared to early/middle adolescence, possibly due to neural re-organization (Uhlhaas et al., 2009; Taylor et al., 2013). The use of broad age ranges in previous studies may decrease sensitivity (De Luca et al., 2003) and mask non-linear development due to the short time-frame when frontal pathways undergo steep maturational change around ages 17–25 (Barker et al., 2010). To address this issue and measure executive function ability across later development, we (Taylor et al., 2013) employed a design with fine-grained age groups (17 years 0 months–17 years 8 months, 18 years 0 months–18 years 8 months, and 19 years 0 months–19 years 8 months) and found non-linear executive function development for strategy generation and concept formation, assessed with D-KEFS Letter Fluency and Sorting Tests (Delis et al., 2001). Seventeen year olds scored significantly higher, indicating better performance, than 18 year olds on strategy generation and four indices of concept formation (number of correct free sorts, free sort description score, sort recognition description score, and description score for perceptual sorts). Seventeen year olds also scored significantly higher, indicating more accurate concept formation than 19 year olds. These findings indicate non-linear executive function development likely reflecting dynamic brain maturation (Lebel et al., 2008; Uhlhaas et al., 2009). Similarly, Dumontheil et al. (2010b) reported non-linear development on a relational reasoning task requiring inhibition and cognitive flexibility (Diamond, 2013) with a dip in accuracy in middle adolescence. Overall these findings indicate that executive functions show linear and non-linear development during adolescence and early adulthood corresponding to linear and non-linear morphological brain changes. Previous studies are limited by cross sectional design so there is a need for longitudinal data to better inform knowledge of cognitive development in late adolescence and early adulthood.

### Social cognition

Imaging studies have consistently implicated a mentalizing network comprised of the medial prefrontal networks, superior temporal sulci, and temporal poles in social cognition task performance (Carrington and Bailey, 2009). Behavioral studies report ongoing social cognition development between adolescence and early adulthood. Vetter et al. (2013) found adolescents aged 12–15 years scored significantly lower than young adults aged 18–22 years on the Story Comprehension Test (Channon and Crawford, 2000), a measure of ToM, and the German version of the Reading the Mind in the Eyes Test (Bölte, 2005), a measure of visual emotion recognition. The development of social cognition was independent of more basic cognitive abilities such as working memory, speed of processing, and verbal ability (Vetter et al., 2013) providing evidence for social cognition being domain specific (Apperly et al., 2005). However, a limitation of the Eyes Test is the use of static stimuli (Baron-Cohen et al., 2001) because they do not fully capture the dynamic and transitory nature of mental states in real life social situations (Vetter et al., 2013). In a longitudinal study, Davis and Franzoi (1991) assessed participants aged 15 and 16 years at 1-year intervals over three consecutive years on the Interpersonal Reactivity Index (IRI; Davis, 1983), a self-report measure of empathy. Perspective Taking, the tendency to consider another person's point of view, and Empathic Concern, the tendency to experience compassion and sympathy toward others, significantly increased, whereas ratings of Personal Distress, the tendency to experience uneasiness in tense social situations, significantly decreased between time points. In contrast, Taylor et al.'s (2013) study showed no group differences in 17, 18, and 19 year olds on social cognition tasks. Previous studies have assessed a narrow range of social cognition so a comprehensive assessment was included in the present study including emotion recognition in visual static stimuli (Reading the Mind in the Eyes Test; Baron-Cohen et al., 2001), auditory stimuli (Reading the Mind in the Voices Test; Golan et al., 2007), dynamic visual and auditory stimuli (Movie for the Assessment of Social Cognition; MASC; Dziobek et al., 2006), and self-report empathy (IRI; Davis, 1983). It is possible that as social cognitive functions are associated with multiple networks (Wolf et al., 2010) that mature earlier than frontal networks, social cognitive functions may be more resistant to change across later development compared to executive functions. Overall, these results highlight the multidimensional nature of social cognition, with different aspects of social cognition showing different developmental trajectories in late adolescence.

To summarize, there is evidence of linear (Romine and Reynolds, 2005; Magar et al., 2010; Vetter et al., 2013) and non-linear development (Dumontheil et al., 2010b; Taylor et al., 2013) of executive functions and social cognition during adolescence. The majority of previous studies examining executive function (Kalkut et al., 2009; Magar et al., 2010) and social cognition (Tonks et al., 2007; Dumontheil et al., 2010a,b; Vetter et al., 2013) in late adolescence and early adulthood have utilized a cross sectional design. This type of design is easier and less time consuming to conduct compared to longitudinal designs (Moriguchi and Hiraki, 2011), although no data on developmental change is collected (Kraemer et al., 2000). The aim of the present study was to investigate the developmental trajectory of executive and social cognitive functions using a longitudinal design with 17 (Younger age group), 18 (Middle age group), and 19 year olds (Older age group) at Time 1 and follow up testing 12–16 months later. Previous research has recommended a longitudinal design to identify whether abilities improve or decline (are linear or non-linear) over time (Romine and Reynolds, 2005; Kalkut et al., 2009). We predicted that executive functions of strategy generation, planning, inhibition, and rule detection would improve and concept formation would decline, based on previous findings, whereas social cognition would be relatively stable between time points.

## Method

All participants gave written informed consent and parental consent was gained for 17 year olds. This research received approval from the Sheffield Hallam University Ethics Committee. A time frame of 12–16 months between testing sessions enabled the identification of any subtle linear and non-linear changes. A minimum 12-month interval between testing sessions conforms to neuropsychological assessment procedure for repeat testing (Lezak et al., 2004) and minimizes memory contributing to practice effects (Hausknecht et al., 2007). Head Injury and Autism Spectrum Disorders were exclusion criteria because of their influence on executive function and social cognition (Dziobek et al., 2006; Robinson et al., 2009; Muller et al., 2010). Participants were recruited from local schools, colleges, youth organizations, and university.

### Participants

Fifty eight participants took part at both time points. Ages of participants in Younger, Middle, and Older groups are presented in Table 1.

**Table 1:** **Means and standard deviations of age for Younger, Middle, and Older groups at Time 1 and Time 2**.

	**Younger group (*n* = 19) 15 females**	**Middle group (*n* = 18) 15 females**	**Older group (*n* = 21) 17 females**
Time 1	*M* = 17 years 4 months *SD* = 2.7 months	*M* = 18 years 4 months *SD* = 2.1 months	*M* = 19 years 2 months *SD* = 2.0 months
Time 2	*M* = 18 years 7 months *SD* = 4.52 months	*M* = 19 years 8 months *SD* = 6.15 months	*M* = 20 years 3 months *SD* = 2.45 months

Participants were asked to report their current Education and changes to living arrangements and friendship groups in the previous 12 months. Seventeen year olds were studying for AS Levels (47%), A2 Levels (47%), and BTEC (Business and Technology Education Council; 6%). Eighteen year olds were studying for A2 Levels (11%), BTEC (11%), and degree (78%) and all 19 year olds were university students.

Changes to living arrangements were highest for 18 and 19 year olds, (72 and 74% respectively) compared to 17 year olds (16%). A higher percentage of 19 year olds (67%) reported making new friends relative to 17 and 18 year olds, (37 and 33% respectively). These data indicate that 18 and 19 year olds had undergone greater change in their living and social environment compared to 17 year olds.

### Procedure

Participants first completed the Positive and Negative Affect Schedule (Watson et al., 1988) to assess mood state and the Hospital Anxiety and Depression Scale (Zigmond and Snaith, 1983) self-report measures to assess anxiety and depression. Participants then completed the Wechsler Abbreviated Scale of Intelligence (Wechsler, 1999) followed by executive function and social cognition tasks which were counterbalanced across testing sessions lasting approximately 3 h. Rest breaks were participant determined. Alternate versions of the D-KEFS Letter Fluency and Sorting Tests were used to ameliorate any testing effects.

### Executive function measures

The executive function battery comprised the D-KEFS (Delis et al., 2001) Letter Fluency Test measure of strategy generation, the Sorting Test measure of concept formation, and Tower Test measure of planning. The Hayling and Brixton Tests (Burgess and Shallice, 1997) provided measures of inhibition and rule detection. The D-KEFS Letter Fluency and Tower Tests were selected because Romine and Reynolds (2005) reported that strategy generation and planning continue to develop between 17 and 22 years. Romine and Reynolds (2005) suggested that future research investigating the development of executive functions should use alternative measures. The D-KEFS Sorting Test was selected as an alternative to the Wisconsin Card Sorting Test (WCST; Heaton et al., 1993) to assess concept formation because there are 16 sorting rules in the D-KEFS version, compared to only 3 in the WCST, increasing task sensitivity and minimizing ceiling effects (Delis et al., 2001). The Hayling Test was selected to assess inhibition because lack of inhibition has been attributed to increased risk taking in this age range (Luna and Sweeney, 2004). The Brixton Test was included to assess rule detection in a spatial format to further explore how this function develops during late adolescence and early adulthood.

### Social cognition measures

Previous studies often focus on one area of social cognition (Vetter et al., 2013) such as empathy (Davis and Franzoi, 1991) or perspective taking (Choudhury et al., 2006; Dumontheil et al., 2010a). The present study assessed various aspects of social cognition using different formats e.g., visual static (Reading the Mind in the Eyes Test; Baron-Cohen et al., 2001), auditory (Reading the Mind in the Voices Test; Golan et al., 2007), dynamic (MASC; Dziobek et al., 2006), and self-report empathy (IRI; Davis, 1983). Tager-Flusberg (2001) conceptualized social cognition as consisting of social-perceptual and social-cognitive processes. The selected tasks support this conceptual framework with the Reading the Mind in the Eyes Test and Reading the Mind in the Voices Test providing measures of social-perceptual processes, whereas the MASC assessed both social-perceptual and social-cognitive processes. The selected tasks support the conceptualization of social cognition as involving processes for understanding others (Eyes Test, Voices Test, and MASC) and understanding the self by including a self-report empathy measure (Beer and Ochsner, 2006).

Table 2 summarizes the executive function and social cognition tasks included in the study.

**Table 2:** **Summary of executive function and social cognition tasks**.

**Task and function measured**	**Function measured**	**Task description**
Hayling and Brixton Tests (Burgess and Shallice, 1997)	Hayling Test (inhibition)	Sentence completion task requiring correct completion (section 1) and inhibition to give an unconnected work (section 2).
	Brixton Test (rule detection)	Predict where a colored circle will move to on the next page.
Delis Kaplan Executive Function System (Delis et al., 2001) Letter Fluency	Strategy generation	Generation of words starting with letters F, A, and S (Time 1) and R, B, and H (Time 2) in 1 min.
Sorting Test	Concept formation	Free sort and sort recognition of cards based on semantic and visuo-spatial features.
Tower Test	Planning	Construct towers from disks whilst only moving one disk at a time and never placing a large disk on a smaller disk.
Reading the Mind in the Eyes Test (Baron-Cohen et al., 2001)	Emotion recognition with visual static stimuli	View photographs of people's eye regions and select one of four complex mental states that best describes how the person is thinking or feeling.
Reading the Mind in the Voices Test (Golan et al., 2007)	Emotion recognition with auditory stimuli	Listen to sound clips and select one of four complex mental states that best describes how the person is thinking or feeling.
Movie for the Assessment of Social Cognition (Dziobek et al., 2006)	Social cognition in dynamic visual and auditory stimuli with social interaction	View film clips and select one of four answers. Task requires consideration of facial expressions, body language, verbal content and intonation.
Interpersonal Reactivity Index (Davis, 1983)	Self-report empathy including empathic concern, personal distress, perspective taking, and fantasy scales	Rate how well statements describe themselves.

### Data analyses

Data were assessed for normal parametric assumptions. Mixed ANOVAs were conducted on IQ, mood, executive function, and social cognition task scores with a between group factor of age group at Time 1 and a within subjects factor of Time 1 and Time 2. Younger, Middle and Older groups refers to participants who were originally in 17, 18, and 19 year old groups at Time 1. Raw scores were analyzed, with the exception of Hayling and Brixton Tests, for ease of comparison across tests because some measures do not have standardized score equivalents. Scaled scores were analyzed for Hayling and Brixton Tests because these are reported extensively in the literature.

## Results

### Participant IQ and mood data

Descriptive statistics for Verbal, Performance and Full Scale IQ, and mood data are presented in Table 3 followed by mixed ANOVAs with age group (Younger, Middle, and Older) as the between group factor and Time 1 and Time 2 as the within group factor.

**Table 3:** **Means and standard deviations for WASI Verbal IQ, Performance IQ, and Full Scale IQ in Younger, Middle, and Older groups at Time 1 and Time 2**.

	**Younger group T1**		**Younger group T2**	**Middle group T1**		**Middle group T2**	**Older group T1**		**Older group T2**
Positive affect	30.28 (4.99)	≈	30.89 (7.59)	30.93 (8.00)	≈	30.67 (6.72)	33.47 (4.22)	≈	33.26 (4.92)
Negative affect	16.00 (6.16)	≈	14.61 (4.83)	14.07 (3.04)	≈	12.60 (2.92)	11.74 (2.49)	≈	13.00 (3.16)
Anxiety	8.83 (2.94)	≈	8.61 (3.55)	7.80 (3.47)	≈	6.13 (3.46)	6.74 (2.45)	≈	7.00 (2.54)
Depression	3.06 (1.77)	≈	3.89 (2.78)	2.07 (1.62)	≈	2.33 (2.69)	3.11 (2.56)	≈	2.37 (2.34)
Verbal IQ	105.00 (7.74)	↑	109.32 (10.69)	103.56(11.16)	↑	109.17 (8.35)	107.90 (6.91)	≈	105.14 (9.19)
Performance IQ	103.16 (12.24)	↑	111.89 (11.08)	99.89(8.70)	↑	106.00 (9.66)	105.57 (7.06)	↑	114.76 (8.50)
Full Scale IQ	104.63 (8.37)	↑	111.89 (10.83)	102.00(10.31)	↑	108.72 (7.93)	107.76 (5.54)	↑	110.95 (6.70)

*Key: ↑ represents significantly better performance at Time 2 relative to Time 1 and ≈ represents no significant change in task scores between Time 1 and Time 2*.

### IQ

Participants varied between Time 1 and Time 2 by −18 to +20 on Verbal IQ, −8 to +25 on Performance IQ and −8 to +18 on Full IQ supporting other reports of variation in IQ during adolescence (Ramsden et al., 2011). All group means fell within the Average range indicating no shift in Verbal IQ category across groups. There was a significant main effect of time [*F*_(1, 55)_ = 5.95, *p* = 0.018] for Verbal IQ score with the Younger [*t*_(18)_ = 2.69, *p* = 0.015] and Middle [*t*_(17)_ = 2.74, *p* = 0.014] groups scoring significantly higher on Verbal IQ at Time 2 compared to Time 1. There was no change for the Older group suggesting that Verbal IQ levels may have stabilized by age 19.

For Performance IQ score there was a significant main effect of time [*F*_(1, 55)_ = 100.25, *p* < 0.001] with Younger [*t*_(18)_ = 5.80, *p* < 0.001], Middle [*t*_(17)_ = 3.71, *p* = 0.002], and Older groups [*t*_(20)_ = 9.09, *p* < 0.001] scoring significantly higher at Time 2 compared to Time 1 on Performance IQ. The mean Performance IQ scores for the Younger and Older groups shifted from Average IQ category at Time 1 to High Average at Time 2.

For Full Scale IQ score there was a significant effect of time [*F*_(1, 55)_ = 61.75, *p* < 0.001] with Younger [*t*_(18)_ = 5.97, *p* < 0.001], Middle [*t*_(17)_ = 4.34, *p* < 0.001] and Older groups [*t*_(20)_ = 3.09, *p* = 0.006] attaining a significantly higher IQ score at Time 2 relative to Time 1. The mean Full Scale IQ score for the Younger group changed from an Average IQ category at Time 1 to High Average at Time 2. A regression was conducted with Performance IQ change score (Time 2–Time 1 score) as a predictor variable and Full Scale IQ change score as the dependent variable to examine how much of the increase in Full Scale IQ was accounted for by improved Performance IQ. This resulted in a significant model [*F*_(1, 56)_ = 24.35, *p* < 0.001] that accounted for 29% of variance (Adjusted *R*^2^ = 0.29) in Full Scale IQ change scores (β = 0.55, *t* = 4.94, *p* < 0.001). Overall IQ findings indicate linear developmental change in Verbal IQ in Younger and Middle groups and linear Performance IQ increase across all age groups indicating that this measure of IQ remains dynamic up to age 20 years and may reflect improved motor skills due to more efficient white matter pathways.

### Mood

There was no significant main effect of time for Positive Affect scores [*F*_(1, 55)_ = 0.07, *p* = 0.788] or Negative Affect scores (*F*_(1, 55)_ = 1.40, *p* = 0.241] of the PANAS (Watson et al., 1988) indicating that mood state was relatively stable across time points for all age ranges. There was no significant main effect of time on Anxiety scores [*F*_(1, 49)_ = 1.53, *p* = 0.222] or Depression scores [*F*_(1, 49)_ = 0.11, *p* = 0.737] from the HADS (Zigmond and Snaith, 1983) for all age ranges indicating that changes in mood did not account for change to other cognitive variables.

#### Executive function measures

Descriptive statistics for executive function task scores are presented in Tables 4 and 5.

**Table 4:** **Means and standard deviations for Younger, Middle, and Older age groups at Time 1 and Time 2 on executive function tasks of inhibition, rule detection, strategy generation, and concept formation**.

	**Younger group**		**Younger group**	**Middle group**		**Middle group**	**Older group**		**Older group)**
	**T1 (*n* = 19)**		**T2 (*n* = 19)**	**T1 (*n* = 18)**		**T2 (*n* = 18)**	**T1 (*****n*** = 21**)**		**T2 (*n* = 21)**
**MEASURES OF RESPONSE INHIBITION AND RULE DETECTION (HAYLING AND BRIXTON TESTS)**									
Hayling scaled	5.84 (1.02)	≈	6.58 (1.26)	5.67 (1.24)	↑	6.50 (0.92)	5.62 (1.36)	↑	6.67 (1.49)
Brixton scaled	7.63 (2.31)	≈	8.47 (1.54)	6.83 (1.98)	↑	7.94 (1.55)	6.86 (1.74)	↑	8.38 (1.43)
**MEASURE OF STRATEGY GENERATION (D-KEFS LETTER FLUENCY TEST)**									
Letter fluency	40.00 (7.88)	↑	43.16 (9.00)	33.28 (8.46)	≈	35.00 (9.40)	37.05 (7.87)	≈	39.05 (9.67)
**MEASURES OF CONCEPT FORMATION (D-KEFS SORTING TEST)**									
Free sorts correct	11.95 (2.12)	≈	11.00 (1.89)	10.72 (1.78)	≈	9.83 (2.09)	10.90 (2.10)	≈	11.10 (2.10)
Free sort description score	45.42 (7.20)	↓	38.68 (7.34)	38.94 (9.43)	≈	36.11 (7.48)	41.90 (7.83)	≈	39.05 (8.88)
Sort recognition description score	50.11 (5.83)	↓	41.79 (6.31)	43.72 (8.46)	≈	41.00 (8.34)	46.33 (7.45)	↓	40.05 (8.93)
Verbal sorts description score	32.16 (8.52)	≈	31.79 (8.53)	26.44 (7.52)	≈	31.00 (7.61)	30.76 (8.11)	≈	31.52 (8.88)
Perceptual sorts description score	63.37 (7.40)	↓	48.68 (8.89)	58.39 (10.81)	↓	46.11 (9.61)	57.43 (8.52)	↓	47.57 (11.50)

*Key: ↑ represents significantly better performance at Time 2 relative to Time 1, ↓ represents significantly poorer performance at Time 2 compared to Time 1, and ≈ represents no significant change in task scores between Time 1 and Time 2*.

**Table 5:** **Means and standard deviations for Younger, Middle, and Older age groups at Time 1 and Time 2 on an executive function task of planning**.

	**Younger group**		**Younger group**	**Middle group**		**Middle group**	**Older group**		**Older group**
	**T1 (/n = 19)**		**T2 (*n* = 19)**	**T1 (/n = 18)**		**T2 (/n = 18)**	**T1 (*****n*** = 21**)**		**T2 (/n = 21)**
**MEASURES OF PLANNING (D-KEFS TOWER TEST)**									
Number of Tower items completed	8.47 (0.91)	≈	8.63 (0.76)	8.11 (1.02)	↑	8.72 (0.58)	8.38 (0.59)	≈	8.71 (0.56)
Tower achievement score	18.16 (3.15)	≈	19.16 (3.39)	18.61 (2.95)	≈	19.33 (3.52)	18.00 (2.85)	↑	20.24 (3.59)
Mean first move time	3.09 (1.14)	↑	2.40 (0.45)	3.94 (2.07)	≈	3.20 (1.46)	4.33 (2.07)	↑	3.25 (1.38)
Time per move	2.51 (0.56)	↑	1.98 (0.27)	2.79 (0.79)	↑	2.31 (0.56)	2.83 (0.52)	↑	2.35 (0.41)
Move accuracy	1.59 (0.37)	≈	1.62 (0.34)	1.60 (0.55)	≈	1.56 (0.28)	1.71 (0.39)	≈	1.59 (0.31)

*Key: ↑ represents significantly better performance at Time 2 relative to Time 1 and ≈ represents no significant change in task scores between Time 1 and Time 2*.

### Response inhibition and rule detection (hayling and brixton tests)

ANOVA results showed a significant main effect of time on the Hayling Test scores [*F*_(1, 55)_ = 20.65, *p* < 0.001]. Results of paired samples *t*-tests showed the Middle [*t*_(17)_ = 3.22, *p* = 0.005] and Older groups [*t*_(20)_ = 3.01, *p* = 0.007] performed better at Time 2, indicating better inhibition, compared to Time 1. There were no other effects. A significant main effect of time was evident on Brixton Test scores [*F*_(1, 55)_ = 28.54, *p* < 0.001] indicating developmental change. Middle [*t*_(17)_ = 3.56, *p* = 0.002] and Older age groups [*t*_(20)_ = 4.36, *p* < 0.001] scored significantly higher at Time 2 compared to Time 1 indicating better rule detection and linear development in these age groups whereas for the younger group these functions remained stable. This suggests ongoing change to these functions may occur later than for Full Scale IQ scores, corresponding well to morphology data and steep maturational peaks at later age.

### Strategy generation (D-KEFS letter fluency test)

A significant main effect of time was found on the Letter Fluency Test indicating developmental change between time points [*F*_(1, 55)_ = 9.25, *p* = 0.004]. The Younger group scored significantly higher at Time 2 compared to Time 1, indicating better strategy generation and linear development [*t*_(18)_ = 2.19, *p* = 0.042]. A significant main effect of age group showed that the Younger group scored significantly higher than the Middle age group [*t*_(35)_ = 2.50, *p* = 0.017], indicating non-linear development and supporting previous findings (Taylor et al., 2013). The older group showed no developmental change between time points indicating that strategy generation matures earlier than other executive functions assessed here and is stable by age 18.

### Concept formation (D-KEFS sorting test)

A significant main effect of time was found on free sort description score [*F*_(2, 55)_ = 9.91, *p* = 0.003], a measure of concept formation, with the Younger group scoring significantly lower at Time 2 relative to Time 1, indicating poorer concept formation and indicative of non-linear development of this function [*t*_(18)_ = 3.68, *p* = 0.002]. The Middle and Older groups showed no developmental change between time points indicating that concept formation, assessed with free sort description score, stabilizes by age 18. No other effects were evident.

Developmental change was evident on the sort recognition description score between time points [*F*_(1, 55)_ = 21.11, *p* < 0.001] with the Younger group scoring lower at Time 2 following a non-linear pattern and indicating poorer concept formation compared to Time 1 [*t*_(18)_ = 4.73, *p* < 0.001]. Similarly, the Older group scored significantly lower on sort recognition description score at Time 2 compared to Time 1 [*t*_(20)_ = 3.15, *p* = 0.005]. There were no other effects.

Description score for perceptual sorts showed developmental change [*F*_(1, 55)_ = 62.96, *p* < 0.001] with the Younger [*t*_(15)_ = 7.51, *p* < 0.001], Middle (*t* (13) = 4.49, *p* = 0.001), and Older groups [*t*_(20)_ = 3.71, *p* = 0.001] scoring significantly lower at Time 2 compared to Time 1, indicating poorer performance and non-linear development. There were no other effects. To summarize, results of analyses indicated developmental change on description score for free sorts, sort recognition and perceptual sorts. These require several executive functions including concept formation, the ability to group cards into categories reflecting a common feature, cognitive flexibility to search for new sorts, and inhibition of repeated sorts (Delis et al., 2001). Overall findings indicate that particular aspects of concept formation are less stable at these age ranges than other executive functions.

### Planning (D-KEFS tower test)

For number of towers completed there was a significant effect of time indicating developmental change in planning ability [*F*_(1, 55)_ = 12.09, *p* = 0.001]. The Middle age group completed significantly more towers at Time 2 relative to Time 1indicating better planning and linear development [*t*_(17)_ = 3.34, *p* = 0.004]. There were no other effects. This suggests a potential spurt in this ability between ages 18 and 19 years that is not seen at younger or older ages.

There was also a significant effect of time on Tower achievement score [*F*_(1, 55)_ = 6.28, *p* = 0.015]. The Older group attained a significantly higher achievement score at Time 2 compared to Time 1 [*t*_(20)_ = 2.16, *p* = 0.043] indicating linear functional development, but there were no other effects. Achievement score takes into account whether towers are completed and the number of moves, indicating that the Older group employed a better planning strategy at Time 2 relative to Time 1.

There was also an effect of time on mean first move on the Tower Test [*F*_(1, 55)_ = 18.74, *p* < 0.001] with the Younger group significantly quicker on first move at Time 2 relative to Time 1 [*t*_(18)_ = 2.47, *p* = 0.024] indicating linear development. A similar pattern was found in the Older group with a significantly shorter mean first move time at Time 2 compared to Time 1 [*t*_(20)_ = 3.73, *p* = 0.001]. No developmental change was found in the Middle group [*t*_(17)_ = 1.73, *p* = 0.102] indicating mean first move time may improve between ages 17 and 18, stabilize between ages 18 and 19, followed by further improvement. There was a significant effect of group for mean first move time [*F*_(2, 55)_ = 3.25, *p* = 0.046] that was investigated further with *post-hoc t*-tests. The Younger group scored significantly lower, showing a faster mean first move time than the Older group [*t*_(31.73)_ = 2.37, *p* = 0.024], indicating non-linear development, with no other group differences evident.

Additionally, there was a significant effect of time on mean time per move scores on the Tower task [*F*_(1, 55)_ = 78.06, *p* < 0.001]. Younger [*t*_(18)_ = 4.74, *p* < 0.001], Middle [*t*_(17)_ = 5.32, *p* < 0.001], and Older groups [*t*_(20)_ = 5.45, *p* < 0.001] showed significantly shorter time per move at the second time point compared to Time 1 indicating linear development of this index of planning.

#### Social cognition measures

Descriptive statistics for social cognition task scores are presented in Table 6.

**Table 6:** **Means and standard deviations for Younger, Middle, and Older age groups at Time 1 and Time 2 on social cognition tasks**.

	**Younger group**		**Younger group**	**Middle group**		**Middle group**	**Older group**		**Older group**
	**T1 (/n = 19)**		**T2 (*n* = 19)**	**T1 (/n = 18)**		**T2 (/n = 18)**	**T1 (*****n*** = 21**)**		**T2 (/n = 21)**
**STATIC VISUAL STIMULI (READING THE MIND IN THE EYES TEST)**									
Eyes	26.63 (5.74)	≈	26.84 (4.65)	27.00 (3.93)	≈	27.00 (3.45)	28.81 (2.44)	≈	28.71 (2.94)
**AUDITORY STIMULI (READING THE MIND IN THE VOICE TEST)**									
Voice	16.53 (2.39)	≈	16.58 (2.87)	16.72 (3.05)	≈	17.33 (2.50)	17.57 (2.16)	≈	17.62 (2.54)
**DYNAMIC VISUAL AND AUDITORY STIMULI WITH SOCIAL INTERACTION (MOVIE FOR THE ASSESSMENT OF SOCIAL COGNITION)**									
MASC correct	35.05 (4.59)	≈	35.00 (6.57)	35.17 (2.64)	↑	36.56 (3.29)	36.19 (6.43)	↑	38.38 (2.31)
MASC excessive errors	5.95 (3.15)	≈	5.26 (3.11)	6.37 (2.03)	↓	5.00 (2.54)	4.90 (2.66)	↓	3.86 (1.68)
MASC insufficient errors	2.74 (2.10)	≈	2.63 (2.41)	2.22 (1.26)	≈	2.50 (1.62)	2.62 (1.63)	≈	1.76 (1.09)
MASC no ToM errors	1.26 (0.93)	≈	1.58 (2.57)	1.22 (0.73)	≈	0.94 (1.11)	1.29 (0.90)	≈	1.00 (0.84)
**SELF-REPORT EMPATHY (INTERPERSONAL REACTIVITY INDEX)**									
IRI Fantasy	19.05 (5.04)	≈	19.42 (3.92)	15.67 (5.51)	≈	14.56 (5.72)	17.90 (5.09)	≈	19.48 (4.42)
IRI Perspective Taking	15.89 (3.56)	≈	17.21 (3.79)	16.83 (4.50)	≈	16.06 (4.71)	18.00 (4.04)	≈	17.81 (3.50)
IRI Empathic Concern	21.21 (3.90)	≈	21.37 (2.61)	20.39 (3.66)	≈	19.39 (4.98)	20.57 (2.73)	≈	21.52 (2.71)
IRI Personal Distress	12.53 (5.27)	≈	10.84 (4.40)	14.61 (5.47)	≈	12.89 (5.93)	13.43 (3.79)	≈	14.52 (5.25)

*Key: ↑ represents significantly better performance at Time 2 relative to Time 1, ↓ represents significantly poorer performance at Time 2 compared to Time 1, and ≈ represents no significant change in task scores between Time 1 and Time 2*.

### Emotion recognition in visual static and auditory stimuli (reading the mind in the eyes and voices tests)

There was no significant effect of time [*F*_(1, 55)_ = 0.01, *p* = 0.915] or group [*F*_(2, 55)_ = 1.75, *p* = 0.183] on the Reading the Mind in the Eyes Test. Similarly, there was no effect of time [*F*_(1, 55)_ = 0.57, *p* = 0.454] or group [*F*_(2, 55)_ = 1.03, *p* = 0.362] on the Reading the Mind in the Voice Test indicating that emotion recognition in visual static and auditory stimuli shows no developmental change beyond age 17.

### Dynamic visual and auditory stimuli with social interaction (movie for the assessment of social cognition)

There was a significant effect of time on total MASC score indicating developmental change [*F*_(1, 55)_ = 5.29, *p* = 0.025], with Middle [*t*_(17)_ = 2.22, *p* = 0.041], and Older [*t*_(20)_ = 3.20, *p* = 0.005] groups scoring significantly higher at Time 2, indicating better social cognition, relative to Time 1, following a linear direction. Similarly, there was an effect of time on MASC excessive mental state inference errors [*F*_(1, 55)_ = 9.73, *p* = 0.003] with the Middle [*t*_(17)_ = 2.38, *p* = 0.029] and Older groups [*t*_(20)_ = 2.36, *p* = 0.029] making significantly fewer errors at Time 2 compared to Time 1 indicating linear improvements and a reduction in over-attribution of mental state content. Finally, there was no effect of time on MASC insufficient mental state inference errors [*F*_(1, 55)_ = 1.15, *p* = 0.288] and MASC no Theory of Mind errors [*F*_(1, 55)_ = 0.13, *p* = 0.718] with no other effects. The finding of Middle and Older groups scoring higher at Time 2 due to fewer excessive mental state inference errors may indicate that social cognition develops between ages 18 and 20 years when assessed with naturalistic, dynamic and auditory stimuli.

### Self-report empathy (interpersonal reactivity index)

There was no effect of time on IRI Fantasy [*F*_(1, 55)_ = 0.25, *p* = 0.618], Perspective Taking [*F*_(1, 55)_ = 0.06, *p* = 0.810], Empathic concern, [*F*_(1, 55)_ < 0.01, *p* = 0.924], and Personal Distress [*F*_(1, 55)_ = 2.52, *p* = 0.118] scales. These findings indicate that self-report empathy is relatively stable by age 17 years.

#### Gender comparisons

Age groups at Time 1 were collapsed and Mann Whitney U tests were conducted to analyse possible gender comparisons between females (*n* = 47) and males (*n* = 11). Results showed a significant difference on two indices of concept formation. Males (*Mdn* = 48.0, range = 18.0) scored higher than females (*Mdn* = 40.0, range = 42.0) on free sorts description score (*U* = 158.50, *z* = 1.99, *p* = 0.047), requiring participants to sort and describe cards. Similarly, males (*Mdn* = 64.0, range = 18.0) scored higher than females (*Mdn* = 59.0, range = 46.0) on description score for perceptual sorts (*U* = 157.00, *z* = 2.02, *p* = 0.044), requiring participants to describe sorts based on visuo-spatial features of cards. There were no other gender group differences on executive function indices (all other *p*s > 0.08).

There were gender group differences on self-report empathy indices of Empathic Concern, sympathetic feelings toward other people's misfortune, and Personal Distress, feelings of apprehension in stressful situations. Females (*Mdn* = 21.0, range = 12.0) scored higher than males (*Mdn* = 19.0, range = 9.0) on Empathic Concern (*U* = 133.00, *Z* = 2.50, *p* = 0.012). Similarly, females (*Mdn* = 14.0, range = 22.0) scored higher than males (*Mdn* = 10.0 range = 12.0) on Personal Distress (*U* = 101.00, *Z* = 3.13, *p* = 0.002). There were no other gender group differences on social cognition tasks (all other *p*s > 0.05). Overall gender analyses indicate that males outperformed females on two indices of concept formation and females outperformed males on two indices of self-report empathy.

Overall, results of longitudinal analyses indicate that executive functions and social cognition follow divergent trajectories. Strategy generation (Letter Fluency Test) improved between ages 17 and 18 followed by no developmental change, whereas inhibition (Hayling Test) and rule detection (Brixton Test) showed later improvement between ages 18 and 20 years. Concept formation (Sorting Test) was less stable than other executive functions with some indices showing non-linear development between time points. Planning (Tower Test) showed evidence of improvements between time points continuing into early adulthood with achievement score, mean first move time and time per move developing between ages 19 and 20. Emotion recognition with static visual stimuli (Eyes Test) and auditory stimuli (Voices Test) and self-report empathy (IRI) showed no development beyond age 17. Social cognition assessed with dynamic stimuli (MASC) showed improvements into early adulthood between ages 18 and 20 years.

## Discussion

The present study extends previous executive function and social cognition research by employing a longitudinal design across peak maturational periods of brain development with narrow age ranges allowing developmental changes to be identified. Participants aged 17, 18, and 19 years at Time 1 completed IQ, executive function and social cognition tasks 12–16 months later (interval between testing *M* = 14.81 months, *SD* = 4.01). We predicted that executive functions of strategy generation, planning, inhibition, and rule detection would improve and concept formation would decline, whereas social cognition would be relatively stable between time points. Results supported the hypotheses with strategy generation improving between ages 17 and 18 years and inhibition and rule detection developing between ages 18 and 20 years. Improvements in planning were evident across age groups on several indices (towers completed improved between ages 18 and 19 years, achievement score improved between 19 and 20 years, mean first move time reduced between ages 17 to 18 years and 19 to 20 years and time per move reduced between time points for all age groups). The hypothesis of concept formation declining was supported by description scores for free sorts, sort recognition, and perceptual sorts declining between time points, indicating non-linear development. The hypothesis of social cognition being relatively stable was partially supported with no development of emotion recognition in visual static and auditory stimuli and self-report empathy beyond age 17 years. Social cognition with dynamic stimuli showed functional improvement between ages 18 and 20 years. Overall these findings indicate that socio-cognitive and executive functions follow divergent developmental trajectories corresponding to divergent brain change based on neural topography. The finding of functions showing improvement or decline at specific ages would not have been captured with broader age ranges as used in other studies. Thus the longitudinal design with fine-grained age groups provided more specific detail about functional developmental change at these ages.

The protracted development of functions into late adolescence and early adulthood may reflect ongoing brain maturation although that is not measured here. Middle and Older age groups scored significantly higher on the Hayling Test at Time 2 compared to Time 1, indicating better inhibition. Section two of the Hayling Test requires inhibition of prepotent responses associated with activation of dorsolateral prefrontal networks (Nathaniel-James and Frith, 2002). Loss of gray matter (via pruning of obsolete cell bodies) commences in dorsolateral prefrontal networks in late adolescence (Gogtay et al., 2004), so the development of cognitive inhibition may reflect synaptic pruning resulting in more efficient neural networks (Sowell et al., 2001). In the present study, the Younger group scored significantly higher at Time 2 on the Letter Fluency Task, indicating better strategy generation, compared to Time 1. There was no developmental change on this measure in the Middle and Older groups indicating that strategy generation stabilizes by age 18. Improved strategy generation in the Younger group may reflect white matter maturation in the Posterior Limb of the Internal Capsule (Bava et al., 2010) specific to this age due to mean diffusivity, an index of white matter integrity, reaching 90% maturation in this brain region by age 18 (Lebel et al., 2008), a similar age to the Younger group at Time 2. All age groups achieved a faster time per move at Time 2 relative to Time 1 on the Tower Test measure of planning. The faster time per move could be explained by ongoing axonal myelination into early adulthood increasing transmission speed (Sowell et al., 2001). Planning tasks require widespread neural networks including frontal, parietal and premotor areas (Wagner et al., 2006), and rapid integration of different neural regions. Greater functional connectivity between these areas could result in more efficient, accurate, and automatic processing (Stevens et al., 2007) evidenced by improved planning indices at Time 2. The finding of divergent executive function developmental trajectories supports the notion of a fractionated executive function system (Miyake et al., 2000).

Present findings showed developmental change on description scores for free sorts, sort recognition and perceptual sorts on the D-KEFS Sorting Test measures of concept formation. Successful performance on these tasks requires participants to consider verbal and perceptual information on sorting cards and the formation of two groups with common attributes whilst concurrently inhibiting previous sorts. According to the manual, higher marks are awarded for more abstract (e.g., warm things and cool things) compared to concrete descriptions (e.g., “you like these on a cold day” and “you like these on a hot day”). Description score for perceptual sorts were significantly lower at Time 2 compared to Time 1, indicating poorer performance, across all age groups indicating non-linear development. This description score is an index of participants' descriptions of visuo-spatial features of the cards (e.g., concave shape vs. convex shape). In addition to the executive functions of concept formation, cognitive flexibility and inhibition, non-executive functions are also measured by the Sorting Test such as perceiving visual features of the cards, use of language and memory. This is a potential problem often highlighted in standardized executive function measures relating to task impurity (Burgess, 1997) because non-executive functions such as language, memory, and visuo-spatial processing are also measured in executive function tasks since these higher-level functions operate across/integrate other lower level functions (Gioia and Isquith, 2004). The present study showed that the Younger group scored significantly lower at Time 2 compared to Time 1 on free sort description score on the D-KEFS Sorting Test supporting the notion of non-linear development in concept formation (Kalkut et al., 2009; Taylor et al., 2013). This is an example of a transitory destabilization of functions during late adolescence / early adulthood due to functional network re-organization (Uhlhaas et al., 2009).

Our findings showed that performance on the Eyes and Voices Tests was not different across time points indicating that emotion recognition of visual static and auditory stimuli is relatively stable across late adolescence and early adulthood supporting previous cross-sectional findings (Taylor et al., 2013). At Time 2, the Middle and Older groups scored significantly higher on the MASC due to fewer excessive mental state inference errors compared to Time 1 indicating that social cognition may develop linearly in late adolescence / early adulthood when assessed with naturalistic, dynamic stimuli. The Eyes and Voice Tests assess social-perceptual aspects of social cognition (Tager-Flusberg, 2001) requiring understanding and interpretation of information from faces, voices, and body posture and mental state attribution. In addition to social-perceptual processes, the MASC is considered to assess social-cognitive processes, the use of information over time and events in the attribution of mental states. The present findings indicate that social-cognitive processes show more protracted development compared to social-perceptual processes, supporting the notion of social-perceptual and social-cognitive components showing different developmental trajectories (Tager-Flusberg, 2001). A possible explanation for the development in MASC scores across time points is the decrease in functional connectivity between adolescence and early adulthood (Burnett and Blakemore, 2009) that could reflect synaptic pruning (Boersma et al., 2011) of unused connections and strengthening of frequently used synapses, resulting in more efficient networks, with a developmental shift from diffuse, extensive activation to focal activation (Durston and Casey, 2006). Imaging studies indicate that performance on the MASC is associated with diverse neural networks including occipito-parietotemporal, temporal and prefrontal networks (Wolf et al., 2010) whereas performance on the Eyes Test in adulthood is associated with activity to the posterior temporal sulcus and inferior frontal gyrus (Moor et al., 2012). Dynamic stimuli are associated with more widespread activation than static stimuli (Trautmann et al., 2009) so it is possible that improvements on the MASC were due to the development of more efficient neural networks and myelination resulting in improved neural transmission (Sowell et al., 2001) between widespread regions.

Present findings of Verbal IQ developing between ages 17 and 19 years and Performance IQ developing between ages 17 and 20 years support the notion that IQ continues to develop into late adolescence and early adulthood (Wechsler, 1981; Ramsden et al., 2011). Verbal IQ means were within the average range so Verbal IQ change cannot account for any other developmental change on executive function and social cognition tasks. The decline in free sort description score, a measure of concept formation between ages 17 and 18 years is in contrast to developments in Verbal IQ indicating that concept formation shows a different developmental trajectory to IQ. All groups scored significantly higher at Time 2 compared to Time 1 on Performance IQ possibly reflecting an improvement in speed of processing due to increased neural transmission and white matter integrity (Sowell et al., 2001).

One issue with longitudinal research is practice effects, better performance on tests due to previous completion and becoming accustomed to the study in general (Jønsson et al., 2006). All groups had a significantly faster mean time per move at Time 2 relative to Time 1 on the Tower Test measure of planning possibly due to participants having completed the task before and already having a strategy to complete the towers. However, practice effects were reduced in the present study by giving participants no feedback about whether answers were correct. An interval of a year between testing minimized memory contributing to practice effects (Hausknecht et al., 2007) and alternative forms of the Letter and Sorting Tests were used at Time 1 and Time 2.

The present study extends previous research by employing a longitudinal design to identify whether abilities improve, decline or stabilize over time (De Luca et al., 2003; Romine and Reynolds, 2005; Waber et al., 2007). It is important to understand the developmental trajectory of functions and whether they show linear or non-linear development. It is of note that the cross sectional data with 17, 18, and 19 year olds (Taylor et al., 2013) and longitudinal analyses are not consistent. For example, no cross sectional group differences were evident on the MASC whereas longitudinal analyses showed that the Middle and Older groups scored significantly higher at Time 2, indicating better social cognition, compared to Time 1. Longitudinal and cross sectional findings are sometimes not consistent because cross sectional analyses show inter-individual (group) differences whereas longitudinal analyses show intra-individual change (Schaie, 2005). Longitudinal analyses may be considered more reliable because in the cross sectional study participants reported considerable changes to living arrangements and friendship groups (Taylor et al., 2013) and Schaie (2005) suggested cross sectional age group comparisons are only appropriate in a stable environment.

The development of social and executive functions may reflect brain maturation and environmental change (Hughes and Ensor, 2009) such as changes to living arrangements and friendship groups (Taylor et al., 2013). Tuvblad et al. (2013) reported that non-shared environmental factors contributed to 54% of variance in Iowa Gambling Test scores at age 16 to 18, indicating that environmental factors influence individual differences in decision making during late adolescence.

There was a gender imbalance with more females taking part in the study than males. Males scored higher than females on two indices of concept formation, free sort description score and description score for perceptual sorts. Females scored higher than males on two indices of self-report empathy, empathic concern, and personal distress, possibly due to social desirability (Laurent and Hodges, 2009). Importantly, results of gender analyses showed relatively few group differences at these age ranges suggesting that development (time) plays a much more important role in the emergence/stability of cognitive functions at these age ranges.

The present findings have educational and clinical implications. Blakemore (2010) proposed that adolescence is a sensitive period for teaching due to protracted neural re-organization and that education should focus on cognitive functions that are still developing. The present results suggest that late adolescence/early adulthood continues to be a sensitive period because some functions show longitudinal development. Sensitive periods can inform educational policy by suggesting at what ages particular skills should be included in the curriculum to optimize learning (Thomas and Knowland, 2009). In a longitudinal study, Miller and Hinshaw (2010) found that executive functions contribute to academic achievement. As longitudinal developmental change was evident on concept formation (Sorting Test), rule detection (Brixton Test) inhibition (Hayling Test) planning (Tower Test), and strategy generation (Letter Fluency Test), perhaps these executive functions could be incorporated more into sixth form and university curricula. Understanding developmental trajectories of functions is important because they have implications for early identification of cognitive dysfunction and treatment outcomes (Kar et al., 2011). Furthermore, the normative longitudinal social and executive function data is relevant in assessing the effectiveness of rehabilitation following Head Injury (Reynolds and Horton, 2008) or in the diagnosis of individuals with Autistic Spectrum Disorders (Brent et al., 2004) and assessing the effectiveness of interventions.

Whilst imaging data is used to explain behavioral changes, future research could combine behavioral and imaging data to map linear and non-linear development of functions onto neural networks. Future research could examine other indices of social cognition task performance such as reaction times. Faster reaction times on tasks with age may reflect increased myelination (Sowell et al., 2001). Appropriate tasks would have dynamic stimuli that show emotional expressions for a short time (Vetter et al., 2013) such as the Movie for the Assessment of Social Cognition or the Cambridge Mindreading Face Voice Battery (Golan et al., 2006). As environmental changes are common in late adolescence and early adulthood, another avenue for future research is to compare social cognition and executive function task scores in participants with constant living arrangements and friendship groups with participants who experience environmental changes.

To conclude, the present longitudinal findings provide further evidence of divergent development of social and executive functions in late adolescence and early adulthood with some functions improving, whilst others decline or stabilize. The protracted development of functions may be attributed to brain maturation including synaptic pruning (Sowell et al., 2001) and functional connectivity (Stevens et al., 2007) and environmental changes (Tuvblad et al., 2013) specific to this age group such as changing friendship groups and living arrangements.

### Conflict of interest statement

The authors declare that the research was conducted in the absence of any commercial or financial relationships that could be construed as a potential conflict of interest.
